# A chimeric porcine reproductive and respiratory syndrome virus 1 strain containing synthetic ORF2-6 genes can trigger T follicular helper cell and heterologous neutralizing antibody responses and confer enhanced cross-protection

**DOI:** 10.1186/s13567-024-01280-3

**Published:** 2024-03-06

**Authors:** Shubin Li, Ming Qiu, Shuai Li, Chen Li, Hong Lin, Yuejia Qiu, Wenhao Qi, Binghui Feng, Meng Cui, Shuai Yang, Wanglong Zheng, Shaobin Shang, Kegong Tian, Jianzhong Zhu, Yu Lu, Nanhua Chen

**Affiliations:** 1 College of Veterinary Medicine, Yangzhou University, Yangzhou, 225009 China; 2 GuoTai (Taizhou) Center of Technology Innovation for Veterinary Biologicals, Taizhou, 225300 China; 3 Joint International Research Laboratory of Agriculture and Agri-Product Safety, Yangzhou, 225009 China; 4 International Research Laboratory of Prevention and Control of Important Animal Infectious Diseases and Zoonotic Diseases of Jiangsu Higher Education Institutions, Yangzhou, 225009 China; 5 Comparative Medicine Research Institute, Yangzhou University, Yangzhou, 225009 China; 6 Jiangsu Co-Innovation Center for Prevention and Control of Important Animal Infectious Diseases and Zoonoses, Yangzhou University, Yangzhou, 225009 China; 7 Key Laboratory of Animal Pathogen Infection and Immunology of Fujian Province, Fuzhou, 350002 China; 8 National Research Center for Veterinary Medicine, Luoyang, 471000 China

**Keywords:** porcine reproductive and respiratory syndrome virus 1 (PRRSV1), ORF2-6 chimeric virus, cross-protection, T follicular helper (Tfh) cells, neutralizing antibodies (nAbs), IFNγ-secreting cells

## Abstract

The prevalence of porcine reproductive and respiratory syndrome virus 1 (PRRSV1) isolates has continued to increase in Chinese swine herds in recent years. However, no effective control strategy is available for PRRSV1 infection in China. In this study, we generated the first infectious cDNA clone (rHLJB1) of a Chinese PRRSV1 isolate and subsequently used it as a backbone to construct an ORF2-6 chimeric virus (ORF2-6-CON). This virus contained a synthesized consensus sequence of the PRRSV1 ORF2-6 gene encoding all the envelope proteins. The ORF2-6 consensus sequence shared > 90% nucleotide similarity with four representative strains (Amervac, BJEU06-1, HKEU16 and NMEU09-1) of PRRSV1 in China. ORF2-6-CON had replication efficacy similar to that of the backbone rHLJB1 virus in primary alveolar macrophages (PAMs) and exhibited cell tropism in Marc-145 cells. Piglet inoculation and challenge studies indicated that ORF2-6-CON is not pathogenic to piglets and can induce enhanced cross-protection against a heterologous SD1291 isolate. Notably, ORF2-6-CON inoculation induced higher levels of heterologous neutralizing antibodies (nAbs) against SD1291 than rHLJB1 inoculation, which was concurrent with a higher percentage of T follicular helper (Tfh) cells in tracheobronchial lymph nodes (TBLNs), providing the first clue that porcine Tfh cells are correlated with heterologous PRRSV nAb responses. The number of SD1291-strain-specific IFNγ-secreting cells was similar in ORF2-6-CON-inoculated and rHLJB1-inoculated pigs. Overall, our findings support that the Marc-145-adapted ORF2-6-CON can trigger Tfh cell and heterologous nAb responses to confer improved cross-protection and may serve as a candidate strain for the development of a cross-protective PRRSV1 vaccine.

**Supplementary Information:**

The online version contains supplementary material available at 10.1186/s13567-024-01280-3.

## Introduction

Porcine reproductive and respiratory syndrome (PRRS) is one of the most economically important swine diseases in pork-producing countries. The aetiologic agent, namely, PRRS virus (PRRSV), is a positive-sense, single-stranded RNA virus grouped within the *Arteriviridae* family [1]. Due to genetic and antigenic diversity, PRRSV isolates are divided into two species: PRRSV1 and PRRSV2 [2]. PRRSV1 was first isolated from the Netherlands in 1991 [3] and can be divided into subtypes 1, 2, and 3 [4]. PRRSV1 isolates are prevalent mainly in Europe at the early stage of emergence but are currently spreading worldwide.

In China, we first reported the isolation of wild-type PRRSV1 [5] and divided Chinese PRRSV1 isolates into four subgroups (Amervac-like, BJEU06-1-like, HKEU16-like and NMEU09-1-like) within subtype 1 [6]. An epidemiological study indicated that the PRRSV1 infection rate reached 24.8% on 50 farms in Guangdong Province [7]. Currently, PRRSV1 infection has been identified in more than 20 provinces [8]. The PRRSV1 SC2020-1 isolate was associated with a 15% abortion rate on a farm in Sichuan Province [9]. More importantly, recent Chinese PRRSV1 isolates (ZD-1, SD1291 and 181,187-2) have been determined to be virulent [8, 10, 11]. However, no specific PRRSV1 vaccine is available in China [12]. Moreover, commercial PRRSV2 vaccines cannot provide satisfactory cross-protection [13, 14].

PRRSV isolates exhibit strict cell tropisms. Primary alveolar macrophages (PAMs) and passaged Marc-145 cells are the most commonly used cells for in vitro PRRSV studies. All commercial PRRSV-modified live vaccines (MLVs) are developed using Marc-145-adapted PRRSV strains. However, all Chinese PRRSV1 field isolates (in addition to PRRSV1 MLV-derived isolates) can be isolated from PAMs but cannot adapt to Marc-145 cells [5, 6, 8, 10, 11, 15], which represents another obstacle to developing a Chinese PRRSV1-specific vaccine.

Several strategies have been utilized to develop cross-protective PRRSV vaccines [16]. Reverse genetics has been frequently employed to improve the heterologous protection of PRRSV vaccines [17, 18]. Our previous study showed that a chimeric HP-PRRSV2 strain containing the PRRSV2 ORF2-6 consensus sequence could confer cross-protection against the NADC30-like PRRSV2 isolate [19]. In addition, minor envelope proteins encoded by ORF2-4 genes and major envelope proteins encoded by ORF5-6 genes play synergistic roles in conferring cross-protection [20]. A recent study determined that the low-virulence HLJB1 isolate could provide limited cross-protection against only the heterologous SD1291 isolate even though they were both clustered within the PRRSV1 BJEU06-1-like subgroup [21].

Therefore, we generated the first infectious clone of the Chinese PRRSV1 isolate in this study and used it as a backbone to construct a chimeric virus containing the Chinese PRRSV1 ORF2-6 consensus sequence. The cross-protective efficacy of this chimeric virus was evaluated and compared with that of the backbone virus in piglets. Clinical signs, viremia, and tissue lesions were recorded during pathological examination. The levels of PRRSV-neutralizing antibodies (nAbs), T follicular helper (Tfh) cells, and IFN-γ-secreting cells were also detected to explore their roles in conferring cross-protection [22–24].

## Materials and methods

### Viruses and cells

SD1291 is a virulent PRRSV1 strain isolated from Shandong Province in 2022 [8]. HLJB1 is a low-virulence PRRSV1 strain isolated from Heilongjiang Province, China, in 2014 [6]. Both the SD1291 and HLJB1 isolates can be passaged only in PAMs. PAMs were harvested from the lung lavage fluid of 6-week-old healthy piglets and cultured in Roswell Park Memorial Institute 1640 medium (RPMI-1640) (HyClone, USA) supplemented with 10% foetal bovine serum (FBS) (EallBio, China), 100 U/mL penicillin and 100 μg/mL streptomycin (Solarbio, China). Marc-145 and BHK-21 cells were cultured in Dulbecco minimum essential medium (DMEM) supplemented with 10% FBS and antibiotics.

### Design and synthesis of the Chinese PRRSV1 ORF2-6 consensus sequence

The “centralized envelope antigens” approach was adopted to study the high genetic diversity of Chinese PRRSV1 isolates [25, 26]. An ORF2-6 consensus sequence was designed based on 14 representative PRRSV1 strains, including 10 Chinese PRRSV1 isolates (Table 1), which contained the most common amino acids at each position of all the envelope proteins [19]. The generated PRRSV1 ORF2-6 consensus sequence was compared with the corresponding regions in representative isolates, and the frameshift mutations were modified to ensure correct expression of all the envelope proteins [27]. The PRRSV1 ORF2-6 consensus sequence was synthesized by the GENEWIZ Company (Suzhou, China).

**Table 1:** **Similarities in the ORF2-6 gene consensus sequence and that in rHLJB1 compared with representative PRRSV1 isolates.**

	Subtype 1												Subtype 2	Subtype 3
	Amervac GU067771^a^	BJEU06-1 GU047344	HKEU16 EU076704	NMEU09-1 GU047345	LV NC_043487	GZ11-G1 KF001144	HeB3 MN927227	HK5 KF287130	HLJB1 KT224385	SD1291 unreleased	KZ2018 MN550991	SC2020-1 MW115431	Westsib13 KX668221	Lena JF802085
Consensus	94.53^b^	91.54	91.36	90.37	93.92	92.43	91.15	91.25	90.93	88.30	87.31	87.77	83.10	85.69
rHLJB1	92.89	88.59	87.38	86.06	92.14	90.37	88.87	87.63	100	86.42	85.92	85.32	81.39	83.56

^a^The GenBank accession number of each isolate.

^b^The percentages (%) of nucleotide identities.

### Construction of PRRSV1-related rHLJB1 and ORF2-6-CON infectious clones

**Figure 1:**
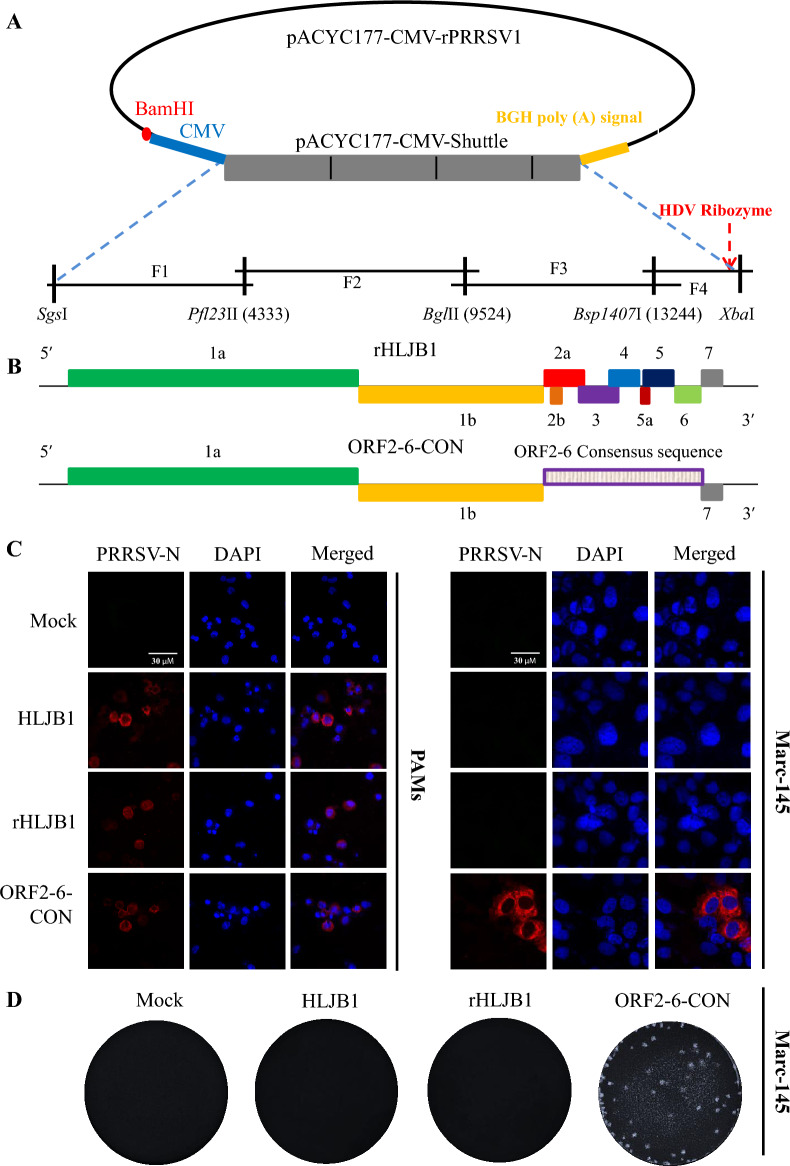
**Design, construction and rescue of the rHLJB1 and ORF2-6-CON infectious clones.** This strategy was adopted from our previous studies [19, 28]. **A** The pACYC177-CMV-stuffer fragment containing five unique restriction enzymes was utilized for the construction of Chinese PRRSV1 full-length cDNA clones. **B** Four overlapping fragments were used to construct full-length rHLJB1 and ORF2-6-CON infectious clones. **C** The successful rescue of rHLJB1 and ORF2-6-CON was identified by IFA in PAMs and Marc-145 cells at 3 dpi, as determined by IFA. **D** The plaque morphology of ORF2-6-CON was determined in Marc-145 cells at 5 dpi.

The construction of a full-length cDNA clone of the PRRSV1 HLJB1 isolate was adapted from our previous study [28]. Briefly, a stuffer fragment containing the CMV promoter, five restriction enzyme sites (*Sgs*I, *Pfl23*II, *Bgl*II, *Bsp1407*I and *Xba*I) and a BGH polyadenylation signal was synthesized and inserted into the low-copy-number pACYC177 plasmid (Suzhou GENEWIZ Company, China). The entire HLJB1 genome was divided into four fragments (Additional file 1A). In the F4 fragment, a copy of the HDV ribozyme sequence was attached immediately after the poly (A) tail through two rounds of PCR [19]. Each fragment was amplified using the primers shown in Table 1. The constructed full-length HLJB1 cDNA clone was confirmed by double digestion with the corresponding enzymes (Additional file 1B) and Sanger sequencing; this clone was named rHLJB1 (Figure 1A).

Overlap PCR was used to construct a chimeric PRRSV1 virus containing the ORF2-6 consensus sequence. First, the primer pair HLJB1-*Bgl*II-F3 + PRRSV1-ORF2-CON-R was used to amplify rHLJB1 to generate the F3-1 fragment. Second, the primer pair PRRSV1-ORF2-CON-F + PRRSV1-ORF6-CON-R was utilized to amplify the synthesized ORF2-6 consensus sequence to generate the F3-F4 fragment. Third, the primer pair PRRSV1-ORF7-CON-F + PRRSV1-Fusion-R was applied to amplify rHLJB1 to generate the F4-2 fragment. All three obtained fragments were treated with *Dpn*I to digest the plasmid templates. Fourth, the primer pair HLJB1-*Bgl*II-F3 + PRRSV1-Fusion-R was utilized for overlap PCR using the F3-1, F3-F4 and F4-2 fragments as templates to generate the F3 + F4 fragment containing the ORF2-6 consensus sequence. Finally, the amplicon was double-digested with *Bgl*II and *Xba*I and subsequently ligated into the rHLJB1 clone to obtain the rHLJB1-ORF2-6-CON clone (abbreviated as ORF2-6-CON) (Figure 1B). The resulting chimeric virus ORF2-6-CON was also confirmed by double enzyme digestion and Sanger sequencing. The complete genome of ORF2-6-CON containing the PRRSV1 ORF2-6 consensus sequence has been submitted to GenBank under accession number OR905562. Both rHLJB1 and ORF2-6-CON were rescued by transfection into BHK-21 cells for 48 h with Lipofectamine 3000 (Invitrogen, USA), after which they were passaged in PAMs or Marc-145 cells [28]. Immunofluorescence assays (IFAs) and confocal microscopy (Leica SP8, Germany) were utilized to evaluate the successful rescue of rHLJB1 and ORF2-6-CON [29, 30]. The PRRSV N-specific murine mAb 15A1 (1:500 dilution) was used as the primary antibody, while DyLight 594 (goat anti-mouse IgG, 1:1000, Invitrogen) was used as the secondary antibody. The plaque morphology was determined in Marc-145 cells as previously described [19].

### Animal experiment

To compare the cross-protective effects of the chimeric ORF2-6-CON strain and the backbone rHLJB1 virus against the heterologous SD1291 isolate, twenty 4-week-old PRRSV-free piglets were divided into four groups (5 pigs per group) and utilized for the inoculation and challenge study. Piglets in Groups 3 and 4 were intramuscularly and intranasally inoculated with 2 mL of 10^4.0^ TCID_50_/mL rHLJB1 (3^rd^ passage) or ORF2-6-CON (3^rd^ passage), respectively. At 42 days post-inoculation (dpi), the piglets in Groups 2, 3 and 4 were challenged with 2 mL of the 10^4.5^ TCID_50_/mL heterologous SD1291 strain (3^rd^ passage), while the piglets in Group 1 were inoculated with RPMI-1640 and used as a mock infection control. The Animal Welfare and Ethics Committee of Yangzhou University approved this animal study with reference number 202209010.

The rectal temperature and clinical symptoms were monitored daily during the first two weeks after inoculation and challenge. Body weight was also determined weekly. Serum samples were acquired weekly for viremia and PRRSV-specific antibody tests. A PRRSV1 universal real-time RT‒PCR assay was used to detect viremia before 42 dpi [31], while SD1291 isolate–specific primers and the TaqMan-MGB probe were utilized to evaluate viremia induced by SD1291 challenge after 42 dpi (Table 2). PRRSV-specific antibodies were identified with a HerdCheck PRRS × 3 ELISA Kit (IDEXX, USA). A sample-to-positive (s/p) ratio of 0.4 was set as the threshold for seroconversion. The sera collected at 0 dpi, 42 dpi (0 days post-challenge (dpc)) and 63 dpi (21 dpc) were also used for virus neutralization tests against HLJB1 and SD1291, as previously described [19, 32]. All the pigs were euthanized at 21 dpc, and tissue samples were collected and utilized for histopathological examination [8]. The gross pathologies of the lungs and lymph nodes were scored in a blinded fashion according to previously described methods [33–35].

**Table 2:** **Primers and probes used in this study for constructing infectious clones and detecting viral loads**

Primers/Probes	Sequence (5 → 3)^a^	Length (bp)
HLJB1-*Sgs*I-F1	AGCTCGGGCGCGCCTACATGATGTGTAGGGTATT	34
HLJB1*-Pfl23*II-R1	ATGGCCTGACTAGGATCGTACGGAACGGCAACCACCGT	38
HLJB1-*Pfl23*II-F2	ACGGTGGTTGCCGTTCCGTACGATCCTAGTCAGGCCAT	38
HLJB1-*Bgl*II-R2	AGCACAGCATCGAGAGGAGATCTACCAGCCCCAACCGGT	39
HLJB1-*Bgl*II-F3	ACCGGTTGGGGCTGGTAGATCTCCTCTCGATGCTGTGCT	39
HLJB1-*Bsp1407*I-R3	ATAAGCAAGTCCGCGTTGTACAAATACGATTCATCGGT	38
HLJB1*-Bsp1407*I-F4	ACCGATGAATCGTATTTGTACAACGCGGACTTGCTTAT	38
1R4	***AGCGAGGAGGCTGGGACCAT***GCCGGCCTTTTTTTTTTTTTTTTTTTTTAATTTCGGTCACATGGTTCCT	69
*XbaI*-2R4	ACAGTCTAGAGTCCCATTCGCCATTACCGAGGGGACGGTCCCCTCGGAATGTTGCCCAGCCGGCGCC***AGCGAGGAGGCTGGGACCAT***^*b*^	87
PRRSV1-ORF2-CON-R	acagtgaccccattgcatcgctccgagttcac^c^	32
PRRSV1-ORF2-CON-F	gtgaactcggagcgatgcaatggggtcactgt	32
PRRSV1-ORF6-CON-R	cttctggctctgatttttaccggccatacttgacg	35
PRRSV1-ORF7-CON-F	cgtcaagtatggccggtaaaaatcagagccagaag	35
PRRSV1-Fusion-R	atggctggcaactagaaggcacag	24
PRRSV1-UF	cagatgcagattgtgttgcct	20
PRRSV1-UR	atggagacctgcagcactttc	20
PRRSV1-UP	FAM-tctggcccctgccca-MGB	14
SD1291-qPCR-F	agatgctgggcgcaatgata	20
SD1291-qPCR-R	ggcttctcaggctttttccttta	24
SD1291-qPCR-P	FAM-ggcggcccagggg-MGB	13

^a^The unique restriction enzymes used for cloning are underlined.

^b^The hepatitis D virus ribozyme sequence is shown in italics, and the overlapping regions are marked in bold.

^c^The primers and probes used for PRRSV1 ORF2-6-CON construction and PRRSV1 detection are shown in lowercase.

### Flow cytometry

We separated mononuclear cells from tissues and blood as previously described [36]. The number of generated mononuclear cells was calculated with a haemocytometer (Sigma‒Aldrich, UK), after which the cells were diluted to a final concentration of 2 × 10^7^ cells/mL. To detect porcine Tfh cells, fresh mononuclear cells were seeded into 96-well V-bottom plates (2 × 10^6^/well). Porcine Tfh cells were stained as previously described [21, 37, 38]. To evaluate the dynamics of IFNγ-secreting T lymphocytes, 2 × 10^6^ cells were seeded in 96-U-bottom-well plates and stimulated with the PRRSV1 heterologous SD1291 strain (multiplicity of infection, MOI = 0.1) for 20 h, while uninfected cells cultured in RPMI-1640 medium were used as a negative control. During the last 5 h of incubation, brefeldin A (Sigma‒Aldrich, USA) was added to the cultures at a final concentration of 5 µg/mL. Subsequently, porcine IFNγ-secreting T cells were stained as we described previously [21]. The stained porcine Tfh cells and IFNγ-secreting T cells were resuspended for FACS analysis. The detailed information on the mAbs used is listed in Additional file 2. A FACS LSRFortessa (BD Biosciences, NJ, USA) was used for flow cytometric analyses. The compensation was determined according to the single-stain samples. A minimum of 200 000 lymphocytes were recorded for analysis of the expression of the transcription factor Bcl-6 and the production of the intracellular cytokine IFN-γ. The obtained data were analysed with FlowJo software (Tree Star, Inc., Ashland, OR, USA). All the gating strategies used were tested with a fluorescence minus one (FMO) control [36].

### Statistical analysis

The rectal temperature, weight gain, viremia, and antibody titre are presented as the means ± standard deviations (SDs). The Mann‒Whitney U test in the GraphPad Prism 8 XML project was used for differential analyses among groups [8, 39]. The statistical significance was set at* p* < 0.05.

## Results

### The PRRSV1 ORF2-6 consensus sequence generally shares increased similarity

Compared with those of representative isolates (LV, WestSib13 and Lena) of the three PRRSV1 subtypes, the ORF2-6 consensus sequence shared increased nucleotide similarity from 92.14 to 93.92%, from 81.39 to 83.10%, and from 83.56 to 85.69%, respectively, compared with that of the backbone rHLJB1 virus. Compared with Chinese PRRSV1 isolates, the ORF2-6 consensus sequence shares > 90% nucleotide similarity with representative strains of four subgroups (94.53% with Amervac, 91.54% with BJEU06-1, 91.36% with HKEU16, and 90.37% with NMEU09-1) and several other isolates (92.43% with GZ11-G1, 91.15% with HeB3, 91.25% with HK5, and 90.93% with HLJB1). Even though the nucleotide similarities were < 90% for the SD1291, KZ2018 and SC2020-1 isolates, the ORF2-6 consensus sequence also had increased nucleotide similarity from 86.42 to 88.30%, from 85.92 to 87.31%, and from 85.32 to 87.77%, respectively, when compared to the rHLJB1 virus. Therefore, the synthetic PRRSV1 ORF2-6 consensus sequence generally shares increased genetic similarity with Chinese PRRSV1 isolates (Table 1).

### PRRSV1 ORF2-6-CON can replicate in both PAMs and Marc-145 cells

The successful rescue of the chimeric strain ORF2-6-CON (containing the ORF2-6 consensus sequence) and the backbone rHLJB1 virus was determined by IFA staining, which showed that PRRSV-specific antigens could be detected in both ORF2-6-CON- and rHLJB1-infected PAMs at 72 h post-infection (hpi) (Figure 1C). Multiple-step growth curves showed that both ORF2-6-CON and rHLJB1 had growth efficacy (relatively lower but not significantly different, *p* > 0.05) similar to that of the parental HLJB1 isolate in PAMs (Additional file 3A). Noticeably, ORF2-6-CON exhibited Marc-145 cell tropism, while HLJB1 and rHLJB1 could not infect Marc-145 cells (Figure 1C and Additional file 3B). In addition, CPE could be observed in only ORF2-6-CON-infected Marc-145 cells at 3–5 dpi (Additional file 4), while plaques were produced in only ORF2-6-CON-infected Marc-145 cells at 5 dpi (Figure 1D). These results indicated that both ORF2-6-CON and rHLJB1 were successfully rescued in vitro, while ORF2-6-CON promoted the adaptation of Marc-145 cells.

### ORF2-6-CON is not pathogenic to piglets

**Figure 2:**
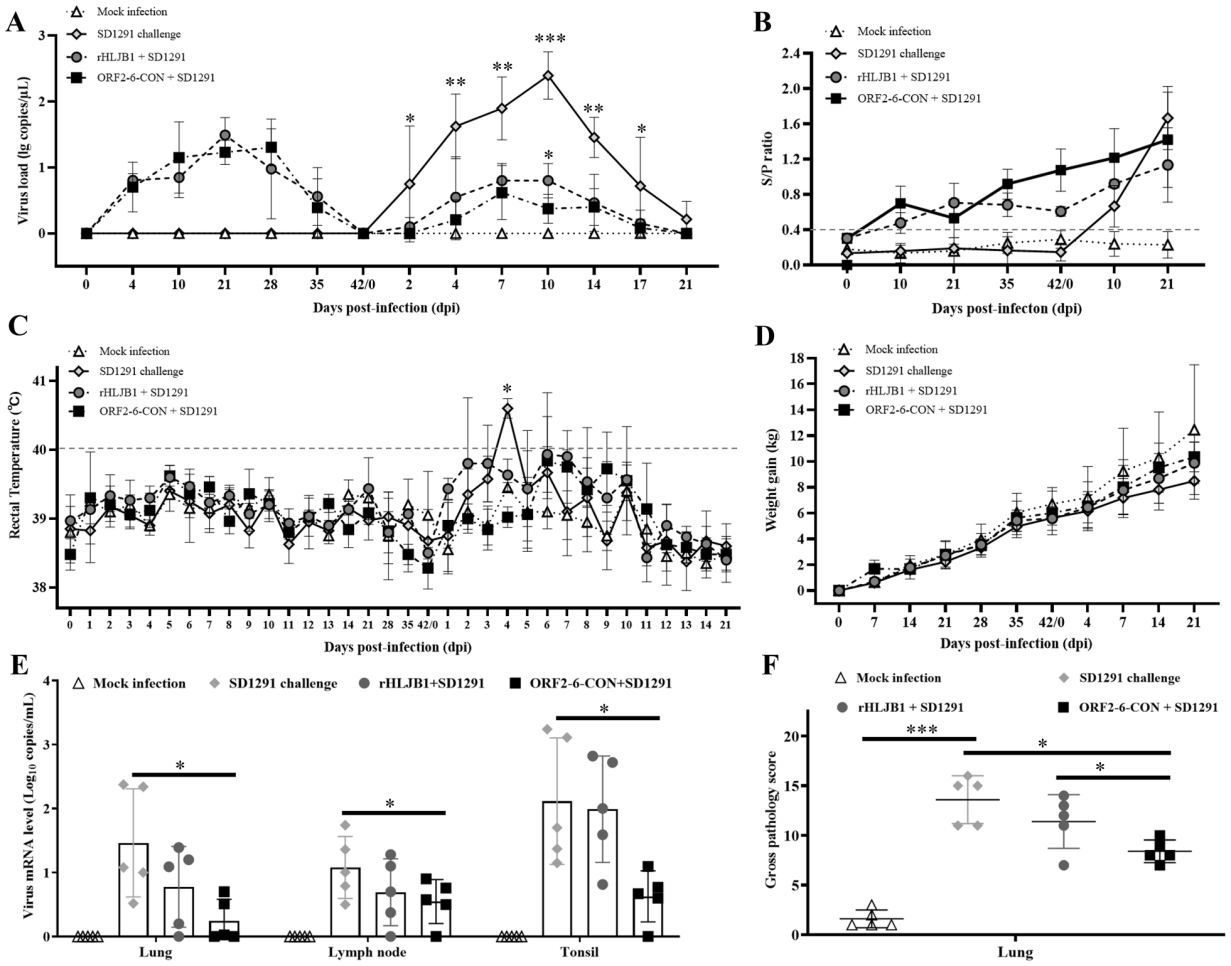
**Dynamics of viral load, antibody level, rectal temperature and weight gain during animal inoculation and challenge study**. **A** Viremia was detected via a PRRSV1 real-time RT‒PCR assay [31]. **B** PRRSV-specific antibodies were detected with an IDEXX HerdCheck PRRS × 3 ELISA Kit. **C** Rectal temperature was determined daily for 14 dpi after inoculation and challenge and weekly thereafter. **D** Body weight was recorded weekly for all groups of pigs. **E** SD1291 viral loads in the lungs, lymph nodes and tonsils among the distinct groups were determined using the SD1291 strain-specific primers and probes shown in Table 2. **F** The gross pathology of the lungs was scored according to methods described previously [33–35]. The data shown in the bar graphs are the means and SDs. Statistical significance is denoted by *, *p* < 0.05; **, *p* < 0.01; and ***, *p* < 0.001 (*n* = 5).

The parental HLJB1 isolate has been determined to have low virulence [21]. In this study, we evaluated the pathogenicity of rHLJB1 and ORF2-6-CON in piglets. Even though inoculation with rHLJB1 or ORF2-6-CON could induce viremia (Figure 2A) and PRRSV-specific antibodies (Figure 2B) within 42 dpi, these infections did not result in any obvious clinical signs, did not cause fever (body temperature < 40 °C, Figure 2C) and did not affect weight gain (Figure 2D). These results suggested that rHLJB1 and ORF2-6-CON are low-virulence strains, similar to their parental virus HLJB1.

### ORF2-6-CON confers enhanced cross-protection against SD1291 challenge

To evaluate the cross-protective effects of rHLJB1 and ORF2-6-CON, pigs in mock-inoculated Group 2, rHLJB1-inoculated Group 3 and ORF2-6-CON-inoculated Group 4 were challenged with the heterologous virulent isolate SD1291 at 42 dpi. After SD1291 challenge, significantly lower viremia was observed in the rHLJB1- or ORF2-6-CON-inoculated pigs than in the SD1291-challenged pigs. In addition, the ORF2-6-CON-inoculated pigs had significantly lower viral loads than the rHLJB1-inoculated pigs at 10 dpc (Figure 2A). Moreover, the SD1291 loads in the lung, lymph node and tonsil were also determined, which showed that the ORF2-6-CON-inoculated pigs had significantly lower viral loads in these tissues than did the SD1291-challenged pigs (*p* < 0.05); moreover, there was no significant difference between the SD1291-challenged pigs and the rHLJB1-inoculated pigs (*p* > 0.05) (Figure 2E). Compared with those in the mock-infected pigs, clinical symptoms (coughing and depression) and fever (> 40 °C) from 3 to 7 dpc appeared in the SD1291-challenged pigs. Moreover, both the rHLJB1- and ORF2-6-CON-inoculated pigs had significantly lower body temperatures at 4 dpc (Figure 2C). Both the rHLJB1- and ORF2-6-CON-inoculated pigs exhibited improved weight gain, which was relatively greater than that of the SD1291-challenged pigs and closer to that of the mock-infected pigs at 21 dpc (not significantly different, *p* > 0.05; Figure 2D).

**Figure 3:**
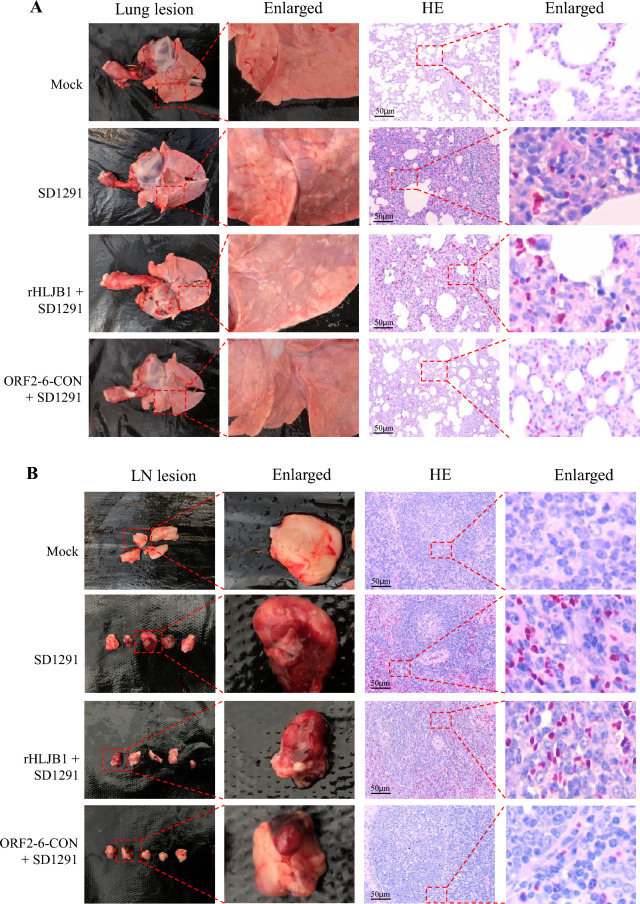
**Post-mortem and histopathological examination.** **A** A representative lung from four groups of pigs collected at 21 dpc. Lung gross lesions and pathological lesions (HEs) in each group were enlarged. **B** Representative hilar lymph nodes from four groups of pigs collected at 21 dpc. Hilar lymph node gross lesions and pathological lesions (HEs) were also enlarged. The enlarged regions of the lungs and hilar lymph nodes (both gross and HE) are shown as red dashed line boxes.

Overall, gross pathology scoring of the lungs revealed that the pigs in the ORF2-6-CON + SD1291 group had significantly fewer pathological lesions than did those in the SD1291-challenged group and the rHLJB1 + SD1291 group (*p* < 0.05) (Figure 2F). Post-mortem examination also revealed lung consolidation and emphysema in the SD1291-challenged group and the rHLJB1 + SD1291 group, while the lungs of the pigs in the ORF2-6-CON + SD1291 group had visibly milder lesions (Figure 3A, Lung lesion). Moreover, interstitial pneumonia with a widened alveolar septum and hyperaemia could be detected in the SD1291-challenged group and the rHLJB1 + SD1291 group, while the histopathological lesions were obviously mitigated in the ORF2-6-CON + SD1291 group (Figure 3A, HE). Similarly, congestion of hilar lymph nodes was detected in three out of five (3/5), 3/5 and 1/5 pigs in the SD1291-challenged, rHLJB1 + SD1291, and ORF2-6-CON + SD1291 groups, respectively (Figure 3B, LN lesion). Histopathological examination revealed hyperaemia and lymphocyte necrosis in hilar lymph nodes from the SD1291-challenged group and the rHLJB1 + SD1291 group, but these changes were obviously alleviated in the ORF2-6-CON + SD1291 group but not in the mock-infected group (Figure 3B, HE). These results suggested that ORF2-6-CON confers greater cross-protection than rHLJB1 against challenge with the heterologous SD1291 isolate.

### ORF2-6-CON induces higher levels of heterologous nAbs and Tfh cells

**Figure 4:**
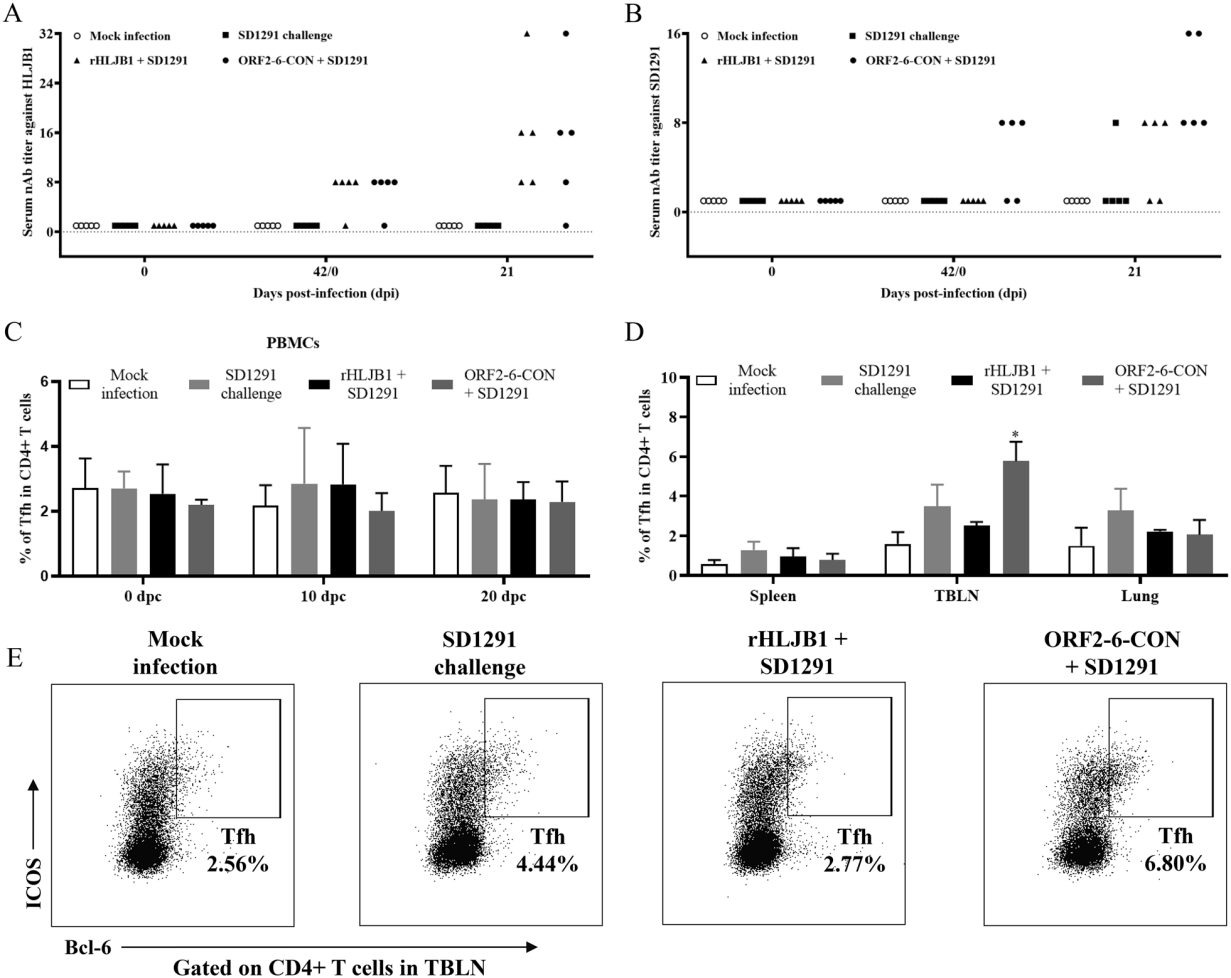
**Generation of nAbs and porcine Tfh cells. A** Generation of nAbs against the HLJB1 strain at 42 dpi and 21 dpc. **B** Generation of nAbs against SD1291 strains at 42 dpi and 21 dpc. **C** The percentages of porcine Tfh cells in blood samples among the four groups at different time points. **D** The percentages of porcine Tfh cells in different tissues among the four groups. * *p* < 0.05. **E** Representative dot plots depicting the percentages of porcine Tfh cells among CD4+ T cells in TBLNs upon SD1291 stimulation (MOI = 0.1). The data are shown as the mean ± SD from 5 pigs per group.

To evaluate the potential roles of protective humoral immune responses in conferring enhanced cross-protection by ORF2-6-CON, the levels of nAbs were determined. At 42 dpi (0 dpc), rHLJB1 and ORF2-6-CON inoculation could induce the same levels of nAbs (1:8 in 4/5 pigs) against the HLJB1 isolate (Figure 4A). However, only ORF2-6-CON-inoculated pigs (3/5) were seropositive for 1:8 of the nAbs against the SD1291 isolate at 42 dpi (Figure 4B). At 63 dpi (21 dpc), similar levels of nAbs against HLJB1 could be induced in both the rHLJB1+SD1291 and ORF2-6-CON + SD1291 groups, while higher levels of nAbs against SD1291 were detected in the ORF2-6-CON + SD1291 group (two pigs with 1:16 nAbs and three with 1:8 nAbs) than in the rHLJB1 + SD1291 group (three pigs with 1:8 nAbs and two with < 1:8 nAbs) (Figure 4B).

Tfh cells are critical regulators of the induction of cross-protective nAbs [24]. Therefore, we analysed the percentages of porcine Tfh cells among CD4 + T cells using the gating strategies shown in Additional file 5A. No significant differences were detected among the blood samples from 0 to 21 dpc (Figure 4C, Additional file 6). Remarkably, a significantly greater percentage of Tfh cells (*p* < 0.05) was detected in tracheobronchial lymph nodes (TBLNs) from ORF2-6-CON + SD1291 pigs (4.64–6.80%) than from the pigs in the other three groups (1.13–2.56% in mock-infected pigs, 2.26–4.44% in SD1291-challenged pigs, and 2.27–2.77% in rHLJB1 + SD1291 pigs) (Fig. 4D, E).

We further explored the potential correlations among Tfh cells, heterologous nAbs and enhanced cross-protection. As shown in Table 3, pigs in the ORF2-6-CON + SD1291 group had higher percentages of Tfh cells in TBLNs, better heterologous nAb responses against the SD1291 isolate, and improved cross-protection. Intriguingly, the no. 18 pig with the highest percentage of Tfh cells in the TBLN (6.80%) also had the highest titre of heterologous nAbs (1:16), a short period of viremia (only detected at 7 dpc) and mitigated lung lesions (Table 3). Taken together, these results indicated that the chimeric strain ORF2-6-CON induces enhanced cross-protective humoral immune responses when compared with the backbone rHLJB1 virus.

**Table 3:** **Responses of Tfh cells, heterologous nAbs and cross-protection among distinct groups**

Groups	Pig no	% of Tfh cells^a^	Heterologous nAbs^b^	Viremia after challenge	Lung lesion
Mock	1	1.39	< 1:8	–	–
	2	2.56	< 1:8	–	–
	3	1.74	< 1:8	–	–
	4	1.13	< 1:8	–	–
	5	1.15	< 1:8	–	–
SD1291 challenge	6	2.26	< 1:8	4–17 dpc	+
	7	4.13	< 1:8	2–21 dpc	+ +
	8	4.44	< 1:8	4–14 dpc	+
	9	4.29	1:8	2–17 dpc	+
	10	2.32	< 1:8	2–21 dpc	+ +
rHLJB1 + SD1291	11	2.77	1:8	2–17 dpc	+
	12	2.35	1:8	4:14 dpc	+
	13	2.27	< 1:8	2–17 dpc	+ +
	14	2.50	< 1:8	4–14 dpc	+
	15	2.61	1:8	4–17 dpc	+
ORF2-6-CON + SD1291	16	5.75	1:8	7–14 dpc	±
	17	6.71	1:16	4–17 dpc	+
	**18**^**c**^	**6.80**	**1:16**	**7 dpc**	** ± **
	19	4.94	1:8	4–17 dpc	+
	20	4.64	1:8	7–14 dpc	+

^a^The percentages of Tfh cells among CD4 + T cells in the TBLN cohort collected at 21 dpc.

^b^The levels of nAb titres against the SD1291 isolate in serum samples collected at 21 dpc.

^c^The no. 18 pig in the ORF2-6-CON + SD1291 group, which has a high percentage of Tfh cells according to the TBLN, a high titre of heterologous nAbs, and improved cross-protection (short viremia period and mild lung lesion), is highlighted in bold.

### ORF2-6-CON and rHLJB1 induce similar SD1291-specific IFN-γ + T-cell levels

**Figure 5:**
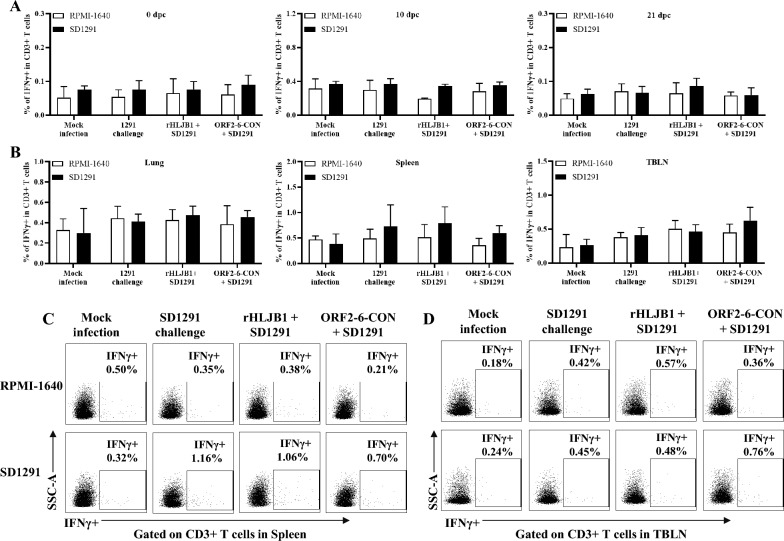
**Frequencies of SD1291-specific IFNγ-secreting T lymphocytes.** **A** Histograms showing IFNγ expression in CD3 + T cells from PBMCs from the four groups after RPMI-1640 or SD1291 stimulation at different time points. **B** Histograms showing IFNγ expression in CD3 + T cells from different tissues in the three groups upon RPMI-1640 or SD1291 stimulation. **C**, **D** Dot plots depicting the percentages of IFNγ-secreting cells among CD3 + T cells from spleens and TBLNs upon stimulation with SD1291. RPMI-1640-incubated cultures were utilized as negative controls. The numbers indicate the percentages of IFNγ-secreting CD3 + T cells. The data are shown as the mean ± SD from 5 pigs per group.

To evaluate the role of cross-protective cellular immune responses in conferring improved cross-protection by ORF2-6-CON, the percentages of IFNγ-secreting cells in blood and tissue samples were also determined using the gating strategies shown in Additional File 5B. Among the peripheral blood mononuclear cells (PBMCs) collected at 0 dpc, 10 dpc and 21 dpc, the percentages of IFNγ-secreting cells among the CD3 + T cells did not significantly differ among the four groups upon stimulation with the SD1291 isolate (Figure 5A). In tissue samples, the percentages of IFNγ-secreting cells in the spleen were relatively higher (*p* > 0.05) in all three PRRSV-infected groups (0.53–1.16% in the SD1291-challenged group, 0.44–1.06% in the rHLJB1+SD1291 group, and 0.55–0.70% in the ORF2-6-CON + SD1291 group) than in the mock-infected group (0.16–0.53%) upon SD1291 stimulation (Figure 5B and C). In TBLNs, a slight increase (*p* > 0.05) in the percentage of IFNγ-secreting cells was observed in the ORF2-6-CON+SD1291 group (0.34–0.76%) compared with the other three groups (0.20–0.36% in the mock-infected group, 0.26–0.45% in the only SD1291-challenged group, and 0.37–0.48% in the rHLJB1 + SD1291 group) (Figure 5B, D). Overall, these results suggested that the chimeric strain ORF2-6-CON confers similar levels of SD1291 strain-specific IFNγ-secreting cells as the backbone rHLJB1 virus.

## Discussion

Due to the high genetic diversity of PRRSV, current vaccines cannot confer satisfactory cross-protection against heterologous isolates [40, 41]. Therefore, a major goal in the design of next-generation PRRSV vaccines is to reduce the genetic dissimilarity between the vaccine strain and the circulating PRRSV isolates to enhance antigenic matching [18]. Reverse genetics has been widely utilized to construct and rescue chimeric vaccine candidates with improved genetic matches for conferring cross-protection. A VR2332-based chimeric virus (JAP56) containing ORF5-6 from JA142 could provide protection against both donor viruses [42]. A chimeric PRRSV strain (VR2385-S3456) containing shuffled ORF3-6 genes could confer enhanced cross-protection against heterologous NADC20 or RFLP 1-7-4 strains in pigs [43]. A PRRSV2 infectious clone virus (PRRSV-CON) with a synthetic consensus genome could induce broader levels of heterologous protection against the 16244B strain than the wild-type FL12 isolate [27]. Here, we constructed and rescued the first Chinese PRRSV1 infectious clone (rHLJB1) and used it as a backbone to generate an ORF2-6 chimeric virus that confers cross-protection against heterologous PRRSV1 isolates.

PRRSV2 was first isolated in China in 1996 and is currently predominant in Chinese swine herds. However, it is easily overlooked that LV-like PRRSV1 (B13) was also detected in China in 1996 [44]. Currently, PRRSV1 isolates have been identified in most provinces in China. Several studies have confirmed that Chinese PRRSV1 wild-type (WT) isolates are pathogenic to pigs [8, 10, 11, 15]. Our previous study showed that the low-virulence HLJB1 isolate cannot provide satisfactory cross-protection against the heterologous SD1291 isolate [21]. Therefore, we evaluated the cross-protective efficacy of a PRRSV1 ORF2-6 chimeric virus (ORF2-6-CON) in this study. Viremia and pathological lesions are two major criteria used to evaluate PRRSV cross-protection [45]. ORF2-6-CON pre-inoculation could reduce SD1291 viremia similar to rHLJB1 pre-inoculation (significantly lower at 10 dpc). In addition, ORF2-6-CON-inoculated pigs presented significantly lower levels of tissue lesions than rHLJB1-inoculated pigs after SD1291 challenge. Thus, ORF2-6-CON provided enhanced cross-protection against the heterologous SD1291 isolate. Whether ORF2-6-CON can also confer cross-protection against other heterologous Chinese PRRSV1 isolates deserves further investigation.

The abundance of these antibodies is significantly correlated with protective immunity against PRRSV [46]. The induction of PRRSV nAbs is a widely acknowledged criterion for evaluating protective humoral immunity [22]. ORF2-6-CON could induce better heterologous nAb responses against the SD1291 isolate than against the rHLJB1 isolate, indicating that heterologous nAbs play a critical role in the cross-protection conferred by ORF2-6-CON. Tfh cells are a specific T-cell lineage that can help B cells differentiate into antibody-secreting cells and play a vital role in regulating the generation of heterologous nAbs [24, 37]. To explore the correlation between porcine Tfh cells and PRRSV1 heterologous nAb responses, we detected porcine Tfh cells in blood and tissue after PRRSV1 inoculation and challenge. Our results showed that the percentage of Tfh cells among CD4 + T cells was significantly greater only in TBLNs from the ORF2-6-CON + SD1291 group. The concurrent generation of Tfh cells and heterologous nAb responses (especially for pig no. 18) suggested that porcine Tfh cells might play a role in inducing heterologous nAbs via ORF2-6-CON. These results are consistent with previous observations in other viruses, such as HIV and SARS-CoV-2, showing that Tfh cells are highly correlated with heterologous nAb responses [24, 47]. Our results provide the first clue for the potential correlation between porcine Tfh cells and heterologous PRRSV nAb responses. However, this finding should be further confirmed by comprehensive investigations using more pigs.

IFNγ-secreting cells also play an essential role in the protective effect of PRRSV [23]. The generation of strain-specific IFNγ-secreting cells is a commonly used criterion for determining protective cellular immune responses [45, 48]. In the present study, relatively more PRRSV-specific IFNγ-secreting cells were detected in the spleens and TBLNs of PRRSV-infected pigs than in those of mock-infected pigs. However, these two subtypes are not significantly different, which might also be associated with the high variation among different pig individuals, delayed T-cell responses and low sensitivity of intracellular cytokine staining [49, 50]. Noticeably, SD1291 strain-specific IFNγ-secreting cells are slightly higher in TBLNs from the ORF2-6-CON-inoculated group than other groups, suggesting that ORF2-6-CON may contain more matched T-cell epitopes with the SD1291 isolate than rHLJB1. Overall, ORF2-6-CON induces similar levels of SD1291-specific IFN-γ-secreting T cells as rHLJB1, suggesting that PRRSV-specific IFN-γ-secreting T cells play a less important role in enhanced cross-protection conferred by ORF2-6-CON.

Intriguingly, ORF2-6-CON exhibited cell tropism towards Marc-145 cells even though the parental HLJB1 and the backbone rHLJB1 viruses cannot replicate in these cells. Although the viral determinants of Marc-145 cell tropism were not identified in this study, further investigations are needed. Even though there is no PRRSV1 commercial vaccine available in China, the cross-protective efficacy of our chimeric strain should be compared with that of other PRRSV1 commercial vaccines in the future. In addition, the immunogenicity of the chimeric virus after serial passage in Marc-145 cells also needs further investigation. Overall, the development of a Chinese PRRSV1-specific vaccine could be vastly facilitated owing to the ability of Marc-145 cells to adapt and enhance the cross-protection conferred by ORF2-6-CON.

This is the first report of the construction and rescue of a Chinese PRRSV1 isolate, which was subsequently used as a backbone for the generation of an ORF2-6 chimeric strain. The chimeric strain ORF2-6-CON could induce better cross-protection than the backbone rHLJB1 virus, which relies mainly on triggering Tfh cell and heterologous nAb responses. This study also provides the first insights into the potential correlations among porcine Tfh cells, heterologous PRRSV1 nAbs and cross-protection. The Marc-145-adapted ORF2-6-CON strain may serve as a promising vaccine candidate strain for combating PRRSV1 infection in China.

### Supplementary Information

**Additional file 1. Construction of the rHLJB1 recombination plasmid.** (A) The four overlapping fragments of the full-length HLJB1 genome were generated by PCR amplification. M: DNA marker, 1-4: F1-F4 fragments of the HLJB1 isolate. (B) The pACYC177-CMV-rHLJB1 recombination plasmid (abbreviated as rHLJB1) was digested with the corresponding digestion enzymes to confirm the successful construction of the full-length cDNA clone of the HLJB1 isolate. M: DNA marker, 1: rHLJB1 without digestion, 2: rHLJB1 digested with SgsI, 3-6: rHLJB1 double-digested with SgsI+Pfl23II, Pfl23II+BglII, BglII+Bsp1407I, and Bsp1407I+XbaI, respectively.

**Additional file 2. Antibodies used in this study.**

**Additional file 3. Multiple-step growth curves for PAMs and Marc-145 cells.** The growth curves within 96 hpi were determined by a PRRSV1 real-time RT‒PCR assay [31].

**Additional file 4. Cytopathic effect (CPE) observation in PRRSV-1-infected Marc-145 cells.** Confluent Marc-145 cells were infected with mock, HLJB1, rHLJB1 or ORF2-6-CON. CPE could be detected in only ORF2-6-CON-infected Marc-145 cells from 3 to 5 dpi.

**Additional file 5. Gating strategies for porcine Tfh cells and IFN-γ-secreting T lymphocytes.** The strategies used were adapted from our previous study [36].

**Additional file 6. Representative dot plots depict the percentages of porcine Tfh cells among CD4+ T cells among PBMCs upon SD1291 stimulation (MOI=0.1).**

## Data Availability

The complete genome of the chimeric virus ORF2-6-CON containing the PRRSV1 ORF2-6 consensus sequence has been submitted to GenBank under accession number OR905562.
