# Supplementary material for: A chimeric porcine reproductive and respiratory syndrome virus 1 strain containing synthetic ORF2-6 genes can trigger T follicular helper cell and heterologous neutralizing antibody responses and confer enhanced cross-protection
Source: Vet Res. 2024 Mar 6;55:28. doi: 10.1186/s13567-024-01280-3 (PMC10918997; doi:10.1186/s13567-024-01280-3)
Supplement: Supplementary file 1 — Additional file 1. Construction of the rHLJB1 recombination plasmid. (A) The four overlapping fragments of the full-length HLJB1 genome were generated by PCR amplification. M: DNA marker, 1-4: F1-F4 fragments of the HLJB1 isolate. (B) The pACYC177-CMV-rHLJB1 recombination plasmid (abbreviated as rHLJB1) was digested with the corresponding digestion enzymes to confirm the successful construction of the full-length cDNA clone of the HLJB1 isolate. M: DNA marker, 1: rHLJB1 without digestion, 2: rHLJB1 digested with SgsI, 3-6: rHLJB1 double-digested with SgsI+Pfl23II, Pfl23II+BglII, BglII+Bsp1407I, and Bsp1407I+XbaI, respectively. [file 13567_2024_1280_MOESM1_ESM.pdf]

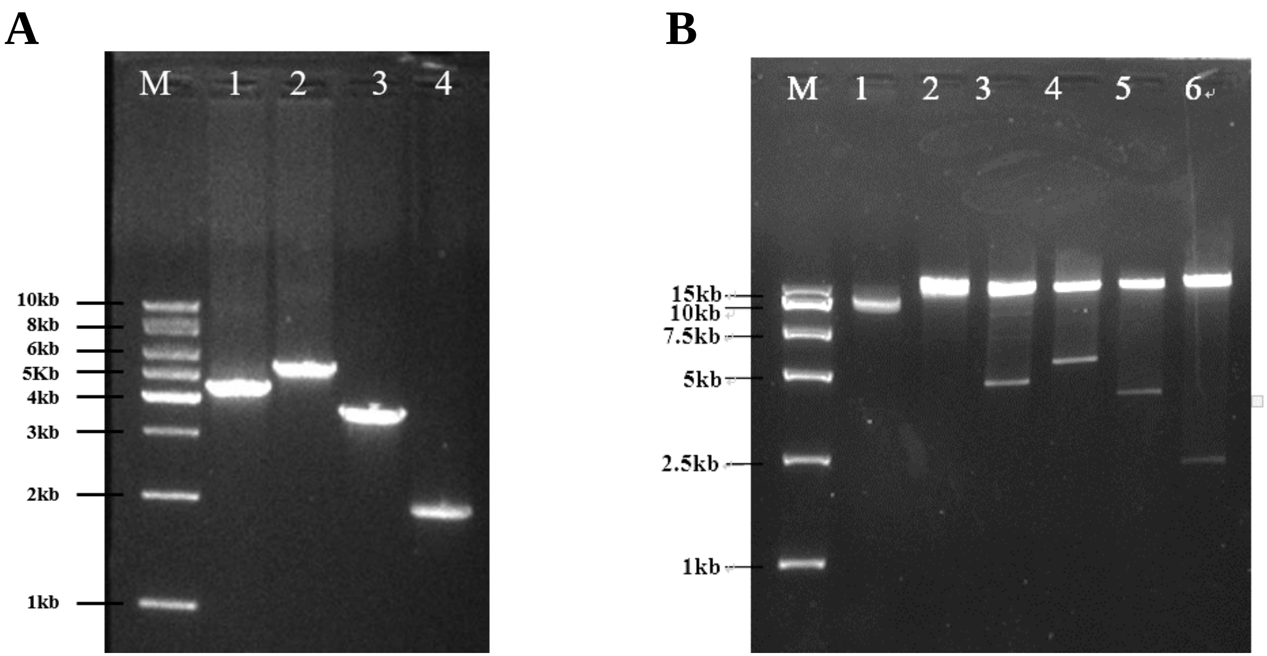

Additional file 1. Construction of rHLJB1 recombination plasmid. (A) The four overlapped fragment of the full-length HLJB1 genome were generated by PCR amplification. M: DNA Marker, 1-4: F1-F4 fragments of HLJB1 isolate. (B) The pACYC177-CMV-rHLJB1 recombination plasmid (abbreviated as rHLJB1) was digested with corresponding digestion enzymes to confirm the successful construction of full-length cDNA clone of HLJB1 isolate. M: DNA Marker, 1: rHLJB1 without digestion, 2: rHLJB1 digested with *Sgs*I, 3-6: rHLJB1 double-digested with *Sgs*I+*Pfl23*II, *Pfl23*II+*Bgl*II, *Bgl*II+*Bsp1407*I, *Bsp1407*I+*Xba*I, respectively.
