# Supplementary material for: A chimeric porcine reproductive and respiratory syndrome virus 1 strain containing synthetic ORF2-6 genes can trigger T follicular helper cell and heterologous neutralizing antibody responses and confer enhanced cross-protection
Source: Vet Res. 2024 Mar 6;55:28. doi: 10.1186/s13567-024-01280-3 (PMC10918997; doi:10.1186/s13567-024-01280-3)
Supplement: Supplementary file 2 — Additional file 2. Antibodies used in this study. [file 13567_2024_1280_MOESM2_ESM.docx]

| Antibodies | Clone | Source |
| --- | --- | --- |
| PE-Cy™7 Mouse Anti-Pig CD4a | 74-12-4 | BD Biosciences |
| PerCP-Cy™5.5 Mouse Anti-Pig CD3ε | BB23-8E6-8C8 | BD Biosciences |
| PE Mouse anti-Bcl-6 | K112-91 | BD Biosciences |
| Brilliant Violet 421™ anti-human/mouse/rat CD278 (ICOS) Antibody | C398.4A | Biolegend |
| Alexa Fluor® 647 Mouse Anti-Pig IFN-γ | P2G10 | BD Biosciences |
| Fixable Viability Dye eFluor™ 780 |  | Thermo Fisher Scientific |
