# Supplementary figures and images for: A chimeric porcine reproductive and respiratory syndrome virus 1 strain containing synthetic ORF2-6 genes can trigger T follicular helper cell and heterologous neutralizing antibody responses and confer enhanced cross-protection
Source: Vet Res. 2024 Mar 6;55:28. doi: 10.1186/s13567-024-01280-3 (PMC10918997; doi:10.1186/s13567-024-01280-3)

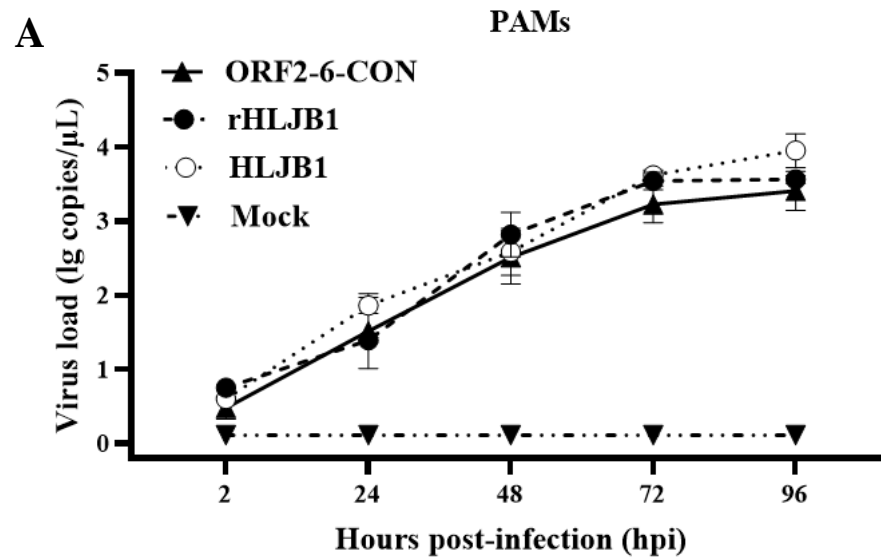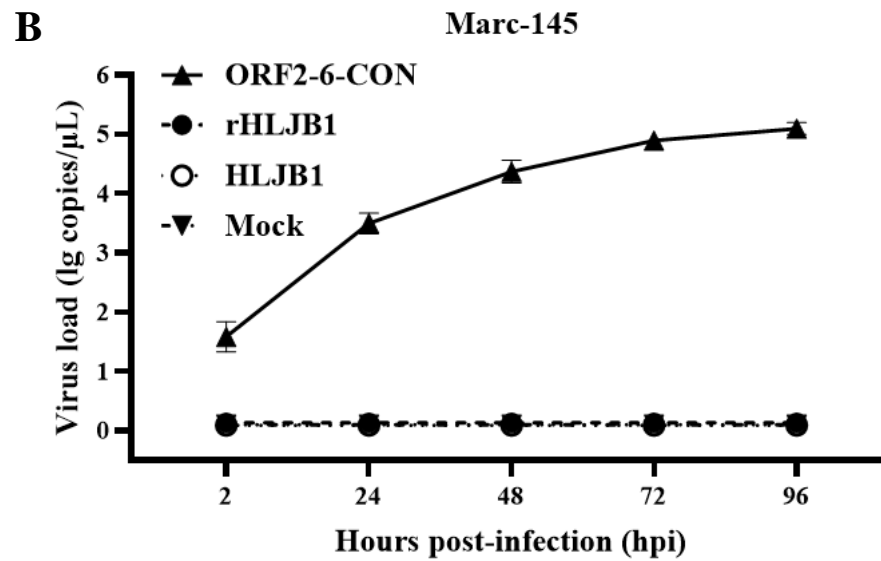

Supplement: Supplementary file 3 — Additional file 3. Multiple-step growth curves for PAMs and Marc-145 cells. The growth curves within 96 hpi were determined by a PRRSV1 real-time RT‒PCR assay [31]. [file 13567_2024_1280_MOESM3_ESM.pdf]

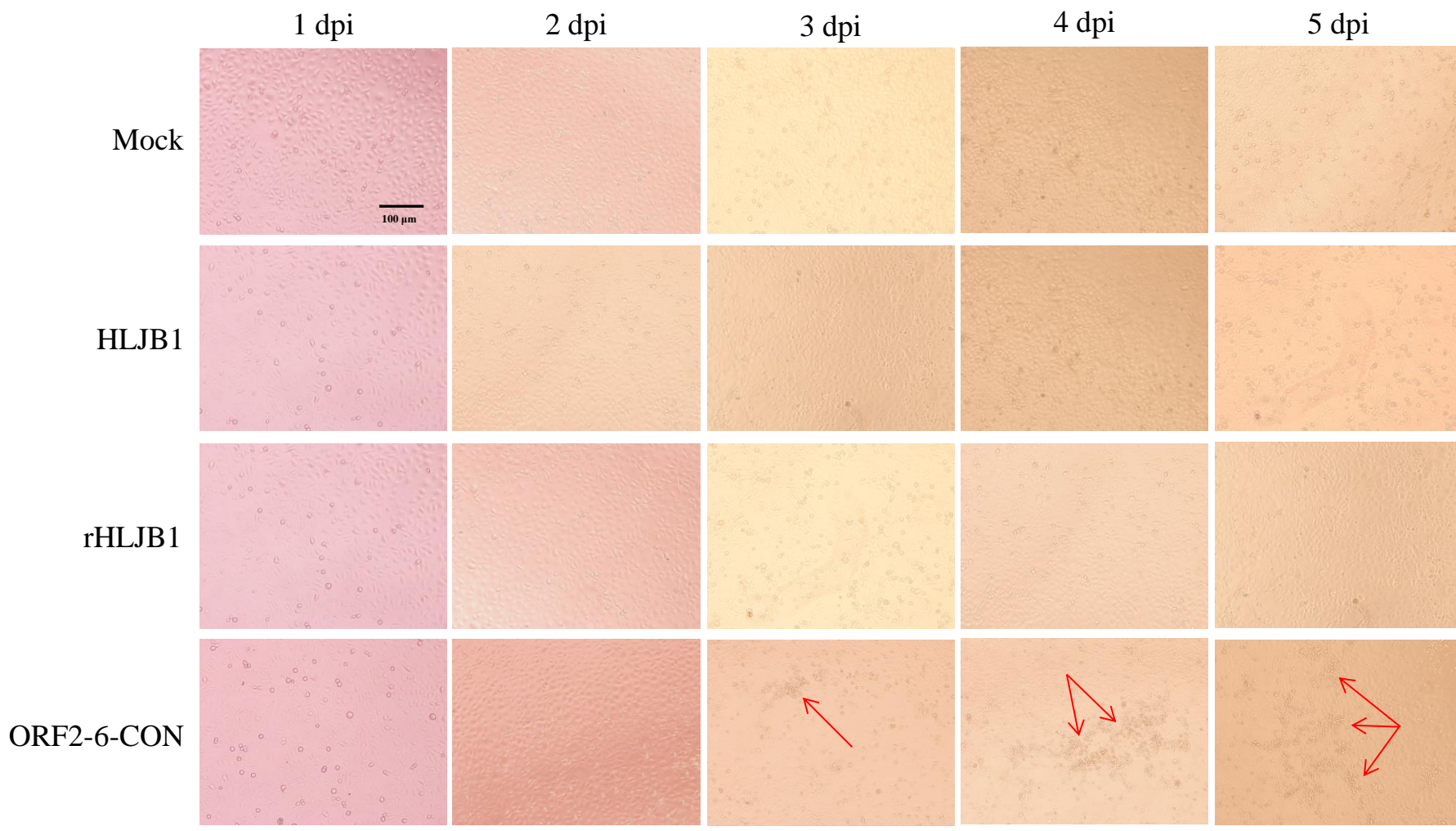

**Marc-145**

Supplement: Supplementary file 4 — Additional file 4. Cytopathic effect (CPE) observation in PRRSV-1-infected Marc-145 cells. Confluent Marc-145 cells were infected with mock, HLJB1, rHLJB1 or ORF2-6-CON. CPE could be detected in only ORF2-6-CON-infected Marc-145 cells from 3 to 5 dpi. [file 13567_2024_1280_MOESM4_ESM.pdf]

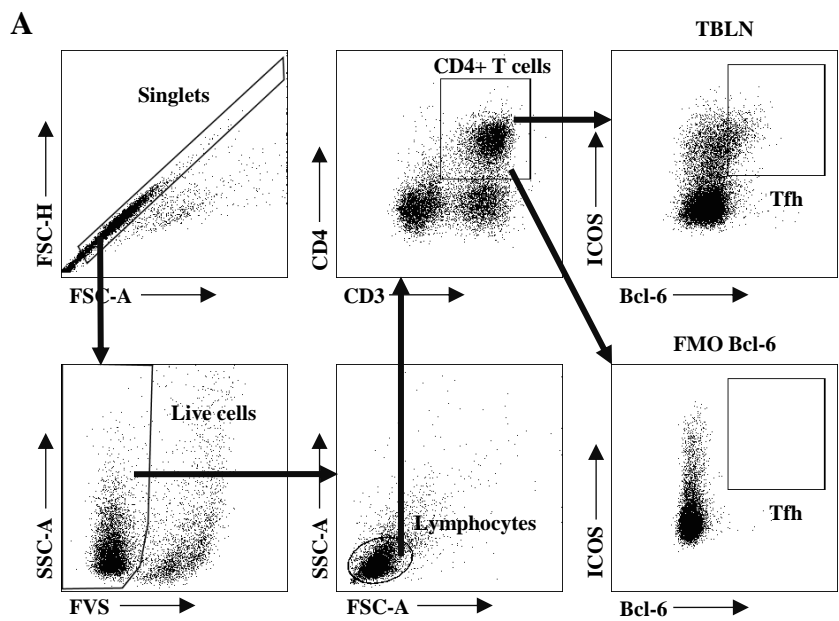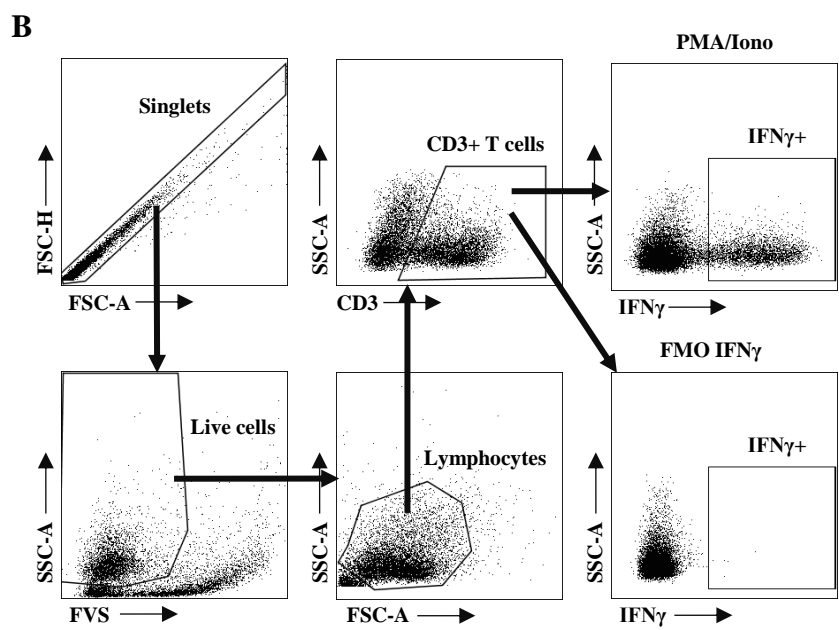

Supplement: Supplementary file 5 — Additional file 5. Gating strategies for porcine Tfh cells and IFN-γ-secreting T lymphocytes. The strategies used were adapted from our previous study [36]. [file 13567_2024_1280_MOESM5_ESM.pdf]

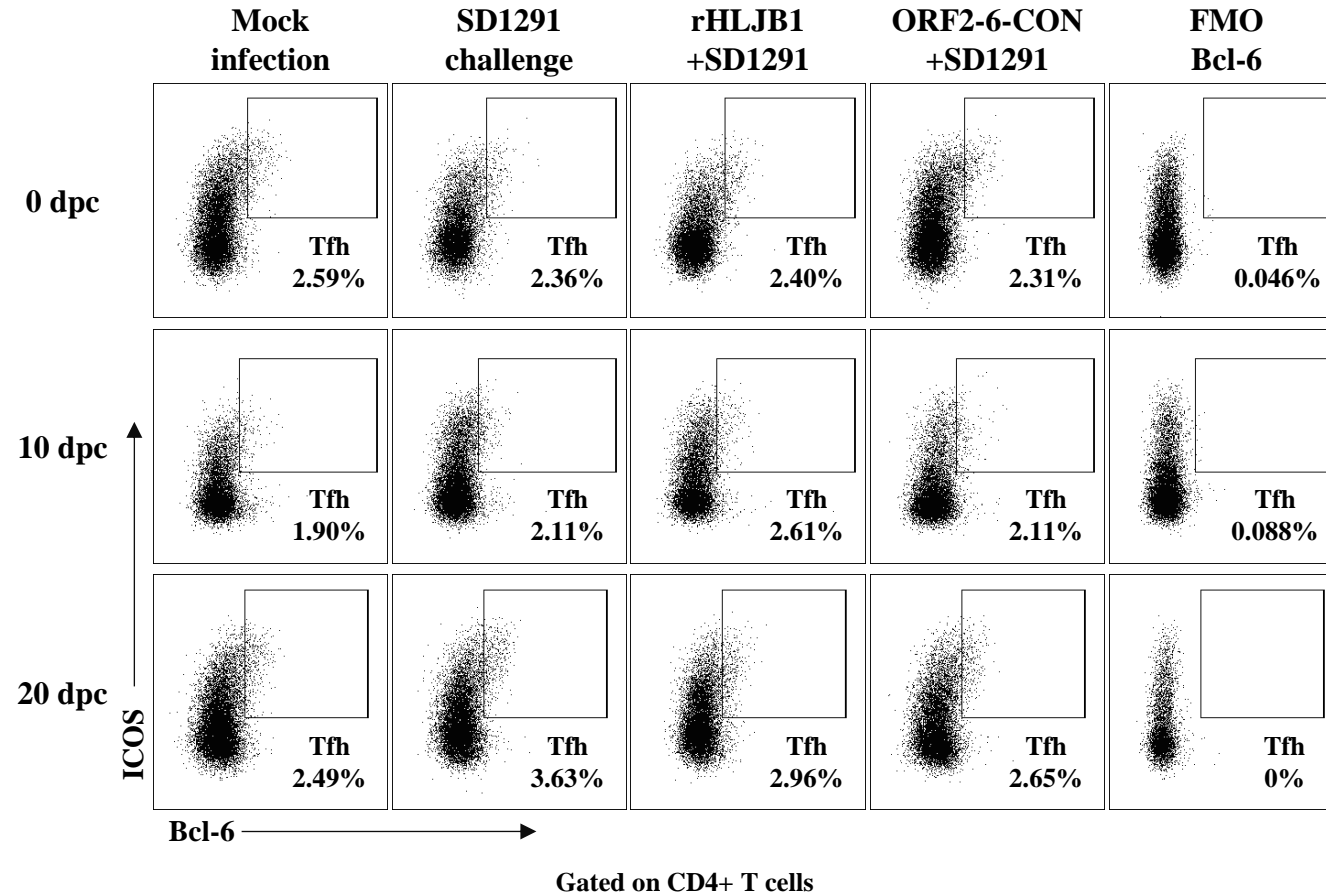

Supplement: Supplementary file 6 — Additional file 6. Representative dot plots depict the percentages of porcine Tfh cells among CD4+ T cells among PBMCs upon SD1291 stimulation (MOI=0.1). [file 13567_2024_1280_MOESM6_ESM.pdf]
